# Diastematomyelia: A Case with Familial Aggregation of Neural Tube Defects

**DOI:** 10.1100/tsw.2004.140

**Published:** 2004-09-21

**Authors:** Nuray Öksüz Kanbur, Pınar Güner, Orhan Derman, Nejat Akalan, Ayşenur Cila, Tezer Kutluk

**Affiliations:** ^1^ Section of Adolescent Medicine, Department of Pediatrics, Hacettepe University Faculty of Medicine, 06100 Ankara, Turkey; ^2^ Department of Public Health, Hacettepe University Faculty of Medicine, Ankara, Turkey; ^3^ Department of Neurosurgery, Hacettepe University Faculty of Medicine, Ankara, Turkey; ^4^ Department of Radiology, Hacettepe University Faculty of Medicine, Ankara, Turkey

**Keywords:** diastematomyelia, neural tube defect, familial aggregation, genetics, pediatrics, Turkey

## Abstract

Intrauterine neural tube defects, meningomyelocele, and diastematomyelia are developmental errors at different stages of the closure of the neural tube. The familial aggregation of these neural tube defects is not previously reported in the literature and should make one think about a common embryogenesis and a possible common mechanism of etiopathogenesis leading to anomalies at different stages of this embryogenesis. This paper presents a 12-year-old Turkish boy with diastematomyelia who was suspected with a demonstrative dermatologic finding without any neurologic sign and diagnosed with magnetic resonance imaging (MRI). He has a positive family history of a stillbirth with neural tube defect, an exitus with meningomyelocele, and an epileptic child in his female siblings.

## INTRODUCTION

Diastematomyelia is a form of spinal dysraphism, defined as sagittal division of the spinal cord into two hemicords, each of which contains a central canal. The etiology is unknown, but it is a congenital malformation as a result of a split notochord. There can be a single dura surrounding them and no spur or fibrous band or two dural tubes with a fibrous band or bony spur within the cleft. It may be associated with some other clinical conditions, other segmental anomalies of the vertebral bodies, and some local dermatologic findings.

This paper presents a 12-year-old Turkish boy with diastematomyelia and tethered cord, who was suspected due to a demonstrative dermatologic finding without any neurologic sign and diagnosed with magnetic resonance imaging (MRI). He had a positive family history of a stillbirth with neural tube defect, a death with meningomyelocele, and an epileptic child in his female siblings. Intrauterine neural tube defect, meningomyelocele, and diastematomyelia are developmental errors at different stages of the closure of the neural tube. The familial aggregation of these neural tube defects is interesting and not previously reported in the literature.

## CASE REPORT

A 12-year-old boy was admitted to the Adolescent Unit with complaint of a congenital hairy patch on his lumbal skin, since birth (see Fig. 1). It was understood that the dimensions of the patch and the length of the hairs were increased by time, necessitating periodic cutting. He had no accompanying symptom except nonspecific lumbalgia. His past medical history was unremarkable. His family history showed that his parents were nonconsanguineous, his mother was 36 years old and one of his sisters was a stillbirth with neural tube defect, one had meningomyelocele and died at age 1 year, and the other sibling was 5 years old with epilepsy. On physical examination, there was no significant finding except a giant lumbal hairy patch, 15 × 24 cm in diameters. His neurological examination was also normal. Blood chemistry and urine analysis were normal. A lumbal spine roentgenogram showed a bony spur at L2 level and fusion defect at L3-4 levels. The lumbal spinal MRI was performed and yielded the diagnosis of "lumbal bony spur and diastematomyelia, tethered cord, L5 stage 1 enterolystesis" (Figs. 2, 3, and 4).

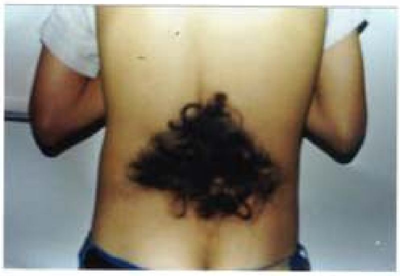

**FIGURE 1.** A congenital hairy patch over the lumbar skin of the patient.

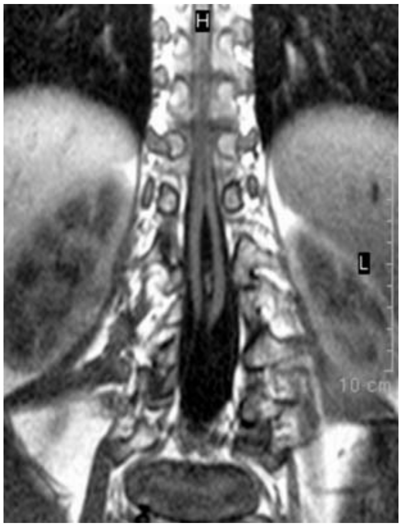

**FIGURE 2.** Coronal T1-weighted image shows diastematomyelia with intervening spur. Note that both hemicords have ended separately. Left sided L2-L3 laminar fusion was also demonstrated.

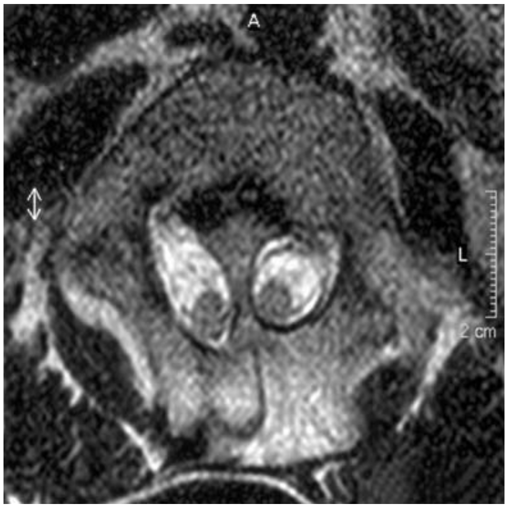

**FIGURE 3.** Axial T2-weighted fast spin echo image showed bony spur dividing both the dural sac and spinal cord.

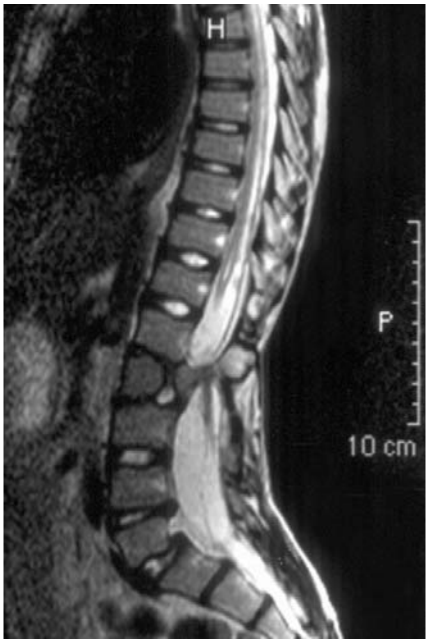

**FIGURE 4.** T2-weighted fast spin echo midsagital image showed bony spur crossing spinal canal at L2 level. Hydromyelia at upper segment was also demonstrated.

## DISCUSSION

A giant, demonstrative hairy patch on the lumbar skin, which continued to expand since birth, was the only symptom in our patient. We suspected an underlying spinal cord pathology since a cutaneous abnormality like hipertrichosis, nevus, skin dimple, or hemangioma on the overlying skin can accompany spinal dysraphysms[1]. Therefore, a lumbal spinal MRI was performed and yielded the diagnosis of diastematomyelia.

Also the family history of our patient made us suspect an underlying neural pathology since he had three siblings with problems (one stillbirth with neural tube defect, another with the diagnosis of meningomyelocele who died at 1 year old, and the third on medication due to epilepsy). Intrauterine neural tube defect, meningomyelocele, and diastematomyelia are developmental errors at different stages of the closure of the neural tube. In the literature, siblings with diastematomyelia have been reported rarely[2], but there was no family reported to have each step of these spinal cord malformations in different siblings. This family is therefore interesting in this aspect.

Diastematomyelia, a congenital splitting of part of the spinal cord, is a rare form of spinal dysraphism. The term “spinal dysraphism” applies to a heterogenous group of spinal abnormalities with the common feature of imperfect formation of the midline mesenchymal, bony, and neural structures. Open dysraphic malformations are the most common type of spinal dysraphism and includes myeloschisis, myelomeningocele, and meningocele. In closed dysraphic malformations, there is no herniation of neural structures or envelopes through the mesenchymal defect. This definition includes the split notocord syndrome, dorsal dermal sinuses, fibrolipomas of the filum terminale, and diastematomyelia[3]. Thus, the diastematomyelia case presented in this report had a closed dysraphic malformation and his sister with meningomyelocele had an open dysraphic malformation and both of them would be common in etiopathogenesis.

A number of seemingly unrelated disorders, including split cord malformations and other complex dysraphic malformations exhibiting disorders of all three primary germ layers are all thought to have a common embryogenesis, variously described as the “endodermal-ectodermal adhesion syndrome”[4]. Disorders of neurulation and caudal neural tube formation include all forms of failure of the neural tube to fuse completely with secondary abnormal development of the mesenchymal structures enclosing the central nervous system, from anencephaly to sacral myelomeningocele and sacral agenesis. In the absence of tube closure, the posterior mesenchyme does not develop, so there is no bony structure covering the neuroectoderm.

The basic anomaly may be nondisjunction of the neural gutter from overlying epithelium, resulting in myelomeningocele or dermal sinus. Premature disjunction could be responsible for lipomyelocele or juxtamedullary or subpial lipoma. This, in turn, could result from a defect in the cell adhesion molecules. Other mechanisms include abnormal canalization or regression of the caudal end of the cord resulting in abnormalities of the filum and abnormal splitting of the notochord responsible for spinal-enteric cysts and diastematomyelia.

Since neural tube closure depends on the complex interaction of multiple cellular processes, it is not surprising that neural tube defects may result from a wide variety of embryonic insults[4]. Neural tube defects have been produced in animals using a number of teratogens, genetic mutations, and experimental manipulations. Although these all suggest a number of possible mechanisms whereby neural tube defects might arise, the cause of human malformations remains unknown[4]. Neural tube defects are most likely etiologically heterogeneous and represent the end result of a variety of embryonic disorders. However, as in the case of the present family, the familial aggregation of these defects should make one think about a common embryogenesis and a possible common mechanism of etiopathogenesis leading to anomalies at different stages of this embryogenesis.

The causes of dysraphism remain obscure, too. They seem to be sufficiently similar to allow all types to be considered together, even though there may be some differences. Genetic factors are important. Usually, they fit to a polygenic or multifactorial model, but a small minority may follow a mendelian type of inheritance with recessive and even X-linked transmission[3]. Environmental factors have been repeatedly stressed, although the search for specific agents has been disappointing.

Recent attention has been focused on the importance of the vitamin folate in the embryogenesis of neural tube defects. Maternal administration of folate antagonists such as aminopterin have long been known to produce neural tube defects[4]. Periconceptional administration of supplemental folate in randomized, placebo controlled studies reduced both the recurrence rate of neural tube defects among women with a previously affected pregnancy and the incidence among women, who had never had an affected pregnancy[5]. However, studies of serum and red blood cell folate levels among mothers of infants with myelomeningocele have produced inconsistent results and folate deficiency does not cause neural tube defects in mice or rat embryos. More recent attention has focused on the possibility that neural tube defects are not caused by absolute folate deficiencies, but rather by abnormalities involving metabolic pathways that require folate — abnormalities that could be overcome by folate supplementation.

Currently, ultrasonography is probably the best tool for antenatal diagnosis for neural tube defects, even for diastematomyelia if performed by a well trained operator. Women who have given birth to a previous fetus with a tube defect should have ultrasonography in a reliable center and given folate supplements regardless of having absolute folate deficiency or not, in addition to genetic counselling.

In the literature, there are some cases of diastematomyelia presented with neurological deficits such as syphincter dysfunction, and bladder and bowel dysfunction with pain, especially in adults. Our patient's neurological examination and urodynamics were totally normal. As their observations confirm that older children and adults with occult spinal dysraphism are more likely to present with irreversible urological and neurological findings than younger children, Satar et al.[6] suggested that it is imperative that a diagnosis should be made and treatment instituted as early as possible. On the contrary, Hood et al.[7] suggested that the presence of diastematomyelia does not automatically mean that the spur must be removed and did not perform surgical treatment to neurologically asymptomatic cases with good follow-up outcomes.

Our patient's work up for diastematomyelia, done by pediatric neurosurgery, consisted of plain lumbar X-rays and MRI study of the spine. Both revealed a bony spur at $L_2$ vertebral level with concomitant $L_2$ vertebral segmentation anomaly. The child was neurologically intact, including bladder function. There was no evidence of scoliosis. The family was informed about the probable course and consequences of tethered cord and a prophylactic surgery was offered. The family decided to continue follow-up at regular intervals instead of surgical intervention.

We decided to share our experience, since pediatricians, obstetricians, and other primary care physicians should be aware of familial aggregation of neural tube defects in order to counsel the family for prenatal-early diagnosis and for prevention modalities such as folate supplements.

## BIOSKETCHES

**Nuray Öksüz Kanbur, MD,** Instructor, Section of Adolescent Medicine, Department of Pediatrics, Hacettepe University Faculty of Medicine, Ankara, Turkey. E-mail: nuraykanbur@hotmail.com

**Pınar Güner, MD,** Resident, Department of Public Health, Hacettepe University Faculty of Medicine, Ankara, Turkey.

**Orhan Derman, MD,** Asistant Professor of Pediatrics, Section of Adolescent Medicine, Department of Pediatrics, Hacettepe University Faculty of Medicine, Ankara, Turkey.

**Nejat Akalan, MD,** Professor of Neurosurgery, Department of Neurosurgery, Hacettepe University Faculty of Medicine, Ankara, Turkey.

**Ayşenur Cila, MD,** Professor of Radiology, Department of Radiology, Hacettepe University Faculty of Medicine, Ankara, Turkey.

**Tezer Kutluk, MD, PhD,** Professor of Pediatrics, Section of Adolescent Medicine, Department of Pediatrics, Hacettepe University Faculty of Medicine, Ankara, Turkey.
